# Quantitative real-time PCR with SYBR Green detection to assess gene duplication in insects: study of gene dosage in *Drosophila melanogaster* (Diptera) and in *Ostrinia nubilalis* (Lepidoptera)

**DOI:** 10.1186/1756-0500-4-84

**Published:** 2011-03-28

**Authors:** Yolanda Bel, Juan Ferré, Baltasar Escriche

**Affiliations:** 1 Department of Genetics, University of Valencia, 46100-Burjassot, Valencia, Spain

## Abstract

**Background:**

The accurate determination of the number of copies of a gene in the genome (gene dosage) is essential for a number of genetic analyses. Quantitative real time PCR (qPCR) with TaqMan detection has shown advantages over traditional Southern-blot and FISH techniques, however the high costs of the required labeled probes is an important limitation of this method. qPCR with SYBR Green I detection is a simple and inexpensive alternative, but it has never been applied to the determination of the copy number of low copy number genes in organisms with high allelic variability (as some insects), where a very small margin of error is essential.

**Findings:**

We have tested the suitability of the qPCR with SYBR Green I detection methodology for the detection of low copy number genes in two insects: the genetically well characterized *Drosophila melanogaster *(Diptera) and the poor genetically characterized *Ostrinia nubilalis *(Lepidoptera). The system was applied to determine the copy number of: (1) the *O. nubilalis cadherin *gene, involved in the mode of action of *Bacillus thuringiensis *toxins, which showed indirect evidence of duplication, and (2) the *D. melanogaster BarH1 *and *BarH2 *genes, located within the *Bar *region of the X chromosome, to clearly determine whether they both are covered by the tandem duplication in the classical *Bar *(*B^1^*) mutant. Our results showed that the *O. nubilalis cadherin *gene is an autosomal single copy gene and that *BarH1*, but not *BarH2*, is duplicated in the Drosophila *B^1 ^*mutant.

**Conclusions:**

This work shows that qPCR with SYBR Green I detection can be specific and accurate enough to distinguish between one and two gene copies per haploid genome of genes with high allelic variability. The technique is sensitive enough to give reliable results with a minimum amount of sample (DNA from individual thoraxes) and to detect gene duplications in tandem.

## Background

Gene duplication is an important evolutionary process that leads to the emergence of gene families and contributes to the evolution of species. In mammals, genetic alteration in gene copy number by amplification or deletion has been defined as one of the main sources of genetic variability [1] and is a common mechanism that leads to deregulation of gene expression and to neoplastic transformation [2,3]. Also, it has been observed that selective gene amplification (repeated gene duplications) is a process that occurs rarely, though spontaneously, in some insects under specific conditions. For example, in *Drosophila*, chorion gene amplification in the ovaries is a well known genetic process that arises to meet the demand for rapid chorion synthesis [4], and in some Hemiptera and Diptera, esterase gene amplification has been described as a molecular mechanism to develop resistance to chemical insecticides [5-7].

The number of copies of a gene per haploid genome (gene dose) has been analyzed traditionally by time-consuming techniques such as Southern blot or fluorescent *in situ *hybridization (FISH). These techniques may not be appropriate when working with insects, either because just small amounts of starting material are available (i.e. when single individual analyses are required) or because of limited resolution (tandem duplications may be missed) [8,9].

The quantitative real time PCR (qPCR following [10]) technique is increasingly adopted for genetic analysis. Two detection chemistries are generally used for this technique: the double-stranded DNA-intercalating agent SYBR Green I dye and the sequence-specific TaqMan probes. The latter has been applied to the analysis of the fruit fly *D. melanogaster *in mapping gene deletions and to determine gene dosage [9], allowing the resolution of two-fold dosage differences in pooled samples of several individuals [11]. SYBR Green I dye detection simplifies the experimental design and reduces the assay costs (there is no need for specific probes), is sensitive [12,13], and yields results comparable to those obtained with the TaqMan chemistry [14]. SYBR Green I may even detect DNA variants present in the target sequence that TaqMan probes would miss [15]. SYBR Green I has been applied to the detection of chorion genes in *Drosophila *(about 30-fold amplification) [4] and to determine the gene copy number of esterase genes (about 30-fold amplification) in insecticide-resistant *Culex *mosquitoes [16]. However, to our knowledge, it has never been applied for determining gene dose in low copy number scenarios, probably because of the possibility to generate false positive results.

The purpose of the present work was to use the qPCR technique with SYBR Green I detection to determine the number of copies of suspected duplicated genes in two insects: the genetically well characterized *Drosophila melanogaster *(Diptera) and the poor genetically characterized *Ostrinia nubilalis *(Lepidoptera).

Mutations in the *cadherin *gene in Lepidoptera have been shown to be linked to resistance to insecticidal proteins from *Bacillus thuringiensis *[17-19]. Because of the continuous increase in the adoption of *Bt*-crops (crops protected from insect attack by expressing insecticidal proteins from *B. thuringiensis*) worldwide, insect resistance to these proteins is of major concern [20]. A very high number of *cadherin *mutant alleles have been found in a *B. thuringiensis *resistant strain of *O. nubilalis *[21]. Several different mutant alleles have also been found in resistant populations of other lepidopteran species such as *Helicoverpa armigera *[22,23] and *Pectinophora gossipyella *[18]. One plausible explanation for the high variability observed is that the *cadherin *locus was duplicated in these lepidopteran species, as was also suspected in a colony of *Plutella xylostella *[24].

On the other hand, the *Bar *mutant (*B^1 ^*strain) of the genetic model species *D. melanogaster *has a cytologically well characterized chromosome tandem duplication segment (region 16A1) which it is responsible for a phenotype with small eyes due to a reduction in the number of ommatidia [25]. The locus *Bar *(*B*) is described in the FlyBase Database [[26], http://flybase.org/reports/FBgn0000154.html] as located on the X chromosome, though it has not been localized to the genome sequence. The FlyBase database describes two *Bar *genes in this chromosome region: the *BarH2 *gene that has the cytological map location 16A1, and the *BarH1 *gene is cytologically located in the region 16A4-16A5. There is no other described gene located in between *BarH1 *and *BarH2 *[[26], http://flybase.org/cgi-bin/gbrowse/dmel/]. In both cases there is only one annotated transcript and the molecular functions described for both genes are the same, as well as the biological processes in which these genes are involved. Interestingly, at present, it is not clear whether the duplicated region in the *B^1 ^*mutant includes both *BarH1 *and *BarH2 *genes or not.

In this study, the qPCR with SYBR Green I chemistry was applied to determine the number of copies of: (1) the *O. nubilalis cadherin *gene, involved in the mode of action of *B. thuringiensis *toxins, which showed indirect evidence of duplication, and (2) the *D. melanogaster BarH1 *and *BarH2 *genes, located within the *Bar *region of the X chromosome, to clearly determine whether they both are covered by the tandem duplication in the classical *Bar *(*B^1^*) mutant. The validity of the technique for such determinations is finally evaluated from the results obtained for both insects.

## Results

### Genetic heterogeneity

**Figure 1:**
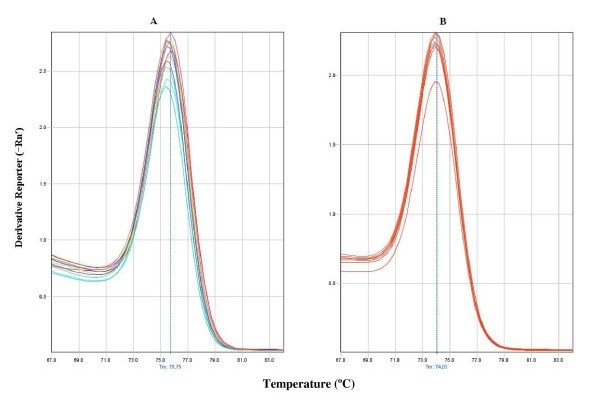
***RpS3 *gene dissociation curves in *O. nubilalis *(A) and *D. melanogaster *(B)**. (A) Dissociation curves of the qPCR *RpS3 *gene amplification products from eight *O. nubilalis *DNA samples (0.1 and 0.01 ng/μl of qPCR reaction). Each DNA sample was obtained from individual thoraxes from insects from the PH, FR or M colonies. Five melting temperature peaks were obtained at 75.75°C, 75.67°C, 75.60°C, 75.52°C and 75.45°C. (B) Dissociation curves of the qPCR products from 7 *D. melanogaster *DNA pooled samples (0.5 and 0.1 ng/μl of qPCR reaction) from thoraxes of 4 adults from the *Or-R *strain. A single melting temperature peak was obtained at 74,03°C.

Variations in sample melting temperatures of the qPCR amplicons in *O. nubilalis *indicated a substantial genetic variability even in the low variability reference gene of the ribosomal protein S3 (*RpS3*). On the contrary, a single melting temperature was found in samples of the genetic pure strains of *D. melanogaster *(Figure 1).

Pure strains are not generally available in Lepidoptera and the samples used in genetic analyses (laboratory colonies or field-derived populations) contain a significant degree of heterogeneity (allelic variation). The genetic variability, when it happens in the central region of the amplicon, affects melting temperatures, producing slight shifts in the peaks. We have observed that when the polymorphism occurs in the nucleotide sequence complementary to the primers, *Ct *values ("threshold cycle value", i.e., the PCR cycle in which a significant increase of the amplified product is first detected) are higher than expected because the incomplete primer's match in the first cycles of amplification lowers the amplification effectiveness and subsequently provokes a delay in the exponential increase of fluorescence (data not shown): this fact influences the GDR (gene dose ratio) values. To overcome this potential problem, we used two sets of primers for each target gene to be studied.

### Gene dose determination in *O. nubilalis*

**Figure 2:**
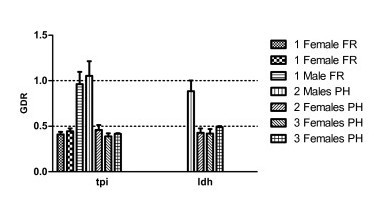
**Gene Dose Ratio of the *tpi *and *ldh *genes in *O. nubilalis***. DNA (0.1 ng/μl) was extracted from 1 or 2 or 3 pooled male or female thoraxes from the PH or the FR colonies. The GDR values are relative to the number of *RpS3 *gene copies in each sample. Error bars represent the SE from three replicates.

The ribosomal protein S3 gene (*RpS3*) was selected as an internal reference single copy gene since it is highly conserved. *RpS3 *has been described in *O. nubilalis *[GenBank: DQ988989] and it is most probably an autosomal single copy gene as are the majority of *Rp *genes in Lepidoptera [27]. In order to confirm this, the *RpS3 *gene was checked with two genes (the lactose dehydrogenase, *ldh *and triosephosphate isomerase, *tpi*) located in the Z sex chromosome [28]. The gene dose ratio (GDR) values obtained are summarized in Figure 2. As can be observed, two sets of values (for one or two gene copies) are clearly separated, with no overlap or intermediate values that could lead to uncertainty. The expected GDR values of around 1 for males (ZZ) and 0.5 for females (ZW) validate the *RpS3 *gene as an autosomal reference gene. In addition, conclusive results can be obtained from samples from a single male or female thoraxes, at different DNA concentrations (from 10 pg to 1 ng DNA/μl, data not shown), and from two different *O. nubilalis *populations (PH and FR).

**Figure 3:**
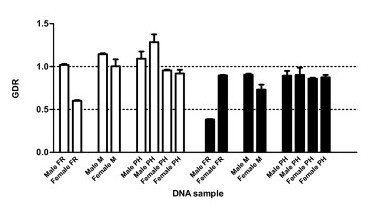
**Gene Dose Ratio of the *cadherin *gene in individual thoraxes of *O. nubilalis***. DNA (0.2 ng/μl) was obtained from individual male or female thoraxes of insects from three *O. nubilalis *colonies. Each DNA sample was analyzed with primers that target exon 26 (e26 primers, empty bars) and exon 28 (e28 primers, filled bars) of the *cadherin *gene. GDR values are relative to the number of *RpS3 *gene copies. Error bars represent the SE from two or three replicates.

Two different target sequences were selected for *cadherin *gene amplification, which are in the exons 26 and 28 (e26 and e28) which code for the putative toxin binding site [29]. Thoraxes of individual males and females from three different colonies (PH, FR and, M) were used and results are summarized in Figure 3. No difference in gene dose was found between males and females from any colony and for either of the two amplified exonic regions of the gene. The only exception was observed in two insects from the FR colony. In the analysis of these individuals, the gene dose value obtained for one of the exons analyzed was close to one as expected, but the amplification of the other exon (e26 in the female and e28 in the male) gave a gene dosage ratio lower than expected. The most plausible explanation is not a difference in gene dose, but an allelic variation in the primer sequences that would affect the amplification of exon 26 in the female and of exon 28 in the male.

In conclusion, the overall data indicate that males and females have the same number of *cadherin *and *RpS3 *gene copies, indicating that *cadherin *is an autosomal single copy gene.

### Gene dose determination in *D. melanogaster*

**Figure 4:**
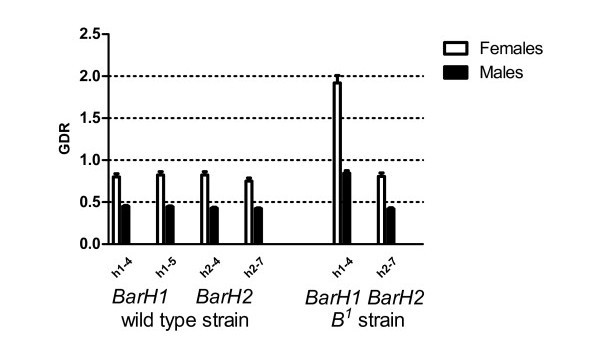
**Gene Dose Ratio of the *BarH2 *and *BarH1 *genes in *D. melanogaster***. DNA samples (0.5 ng/μl) were obtained from pooled thorax samples of males or females from wild type (*Or-R*) and mutant *B^1 ^*strains. GDR values are relative to the number of *RpS3 *gene copies in each sample. The primer pairs used for each gene amplification are indicated below the bars. Error bars represent the SE from three replicates.

The analysis of *BarH2 *and *BarH1 *genes in flies from the wild strain of *D. melanogaster Or-R *provided GDR values in females approximately twice as high as those of males (Figure 4). These results were expected since *Bar *genes are located in the X chromosome and males are the heterogametic sex (XY), females are the homogametic sex (XX) and the GDR values were obtained using an autosomal (AA) gene as a reference. The results were reproducible with two different primer pairs and at different DNA concentrations (from 20 pg to 2.5 ng DNA/μl, data not shown).

The number of copies of the *BarH2 *and the *BarH1 *genes was determined in males and females of the *Bar *mutant *B^1^*, which carries a putative duplication of the *Bar *locus. Gene doses revealed a different gene dose for the two *Bar *genes tested (Figure 4): the *BarH2 *GDR was the same as in wild type insects, while the *BarH1 *gene dose was twice as much, both in males and females, with respect to wild type values. The GDR values were irrespective of the concentration of DNA used in the reaction. These results indicate that the *BarH1 *gene is included in the duplicated region responsible for the *Bar *phenotype, but the *BarH2 *gene is not included.

## Discussion

*O. nubilalis *is an important lepidopteran pest of maize which is well controlled with *Bt*-maize and for which there is a great interest to evaluate its potential to develop resistance to *B. thuringiensis *insecticidal proteins [20]. The *cadherin *gene is one of the candidate resistance genes [17-19] and the high allelic variability observed in Bt-resistant strains in different lepidopteran species [18,22,23], including *O. nubilalis *[21], suggests a gene duplication in the lepidopteran species that only would become detectable in individuals carrying mutations in this gene. Our results showing that the *cadherin *gene in *O. nubilalis *is a single copy gene, discard such a possibility, and are in agreement with Baxter *et al*. [24], who suggested the absence of a *cadherin *gene duplication in *Plutella xylostella *based on the conventional Southern-blot technique. However, these authors were not able to discard a gene tandem duplication because of the limitation of the methodology. The determination of the number of gene copies of this resistance-gene is of importance to design appropriate resistance management strategies to preserve the efficacy of *Bt*-crops.

An interesting result from our study in *O. nubilalis*, is the heterogeneity of melting temperatures in the dissociation curves obtained with samples from individual insects, which reflects the polymorphism of gene sequences within colonies (Figure 1). This had been previously observed with DNA from cancer samples [30] and with virus variants [15] and can be a source of additional information about the number and frequency of alleles in the studied population. In fact, differences in the dissociation temperature of PCR products have been used to distinguish between alleles of the *estβ *gene in *Culex *[16]. We have observed that when the polymorphism affected the nucleotide sequence complementary to the primers, *Ct *values were higher than expected. Therefore, in colonies that have not been highly inbreed, due to the possibility of sequence variability, it is advisable use at least two primer pairs for each target gene to ensure the validity of the *Ct *values obtained.

*Bar *(*B*) is a *D. melanogaster *developmental mutant of the imbalanced gene dosage type. In the *B^1 ^*mutant, the precise limits of the duplication have never been molecularly investigated, even though this is a classical mutant described in the beginnings of Genetics in the early 20th Century [25]. This uncertainty is probably due to the technical difficulties to study identical gene copies in tandem. The present results has permitted to accomplish precise molecular mapping showing that the duplication in the *B^1 ^*mutant affects only one gene (*BarH1*) within the *Bar *region.

The results obtained in the present work show the capacity of the qPCR technique with SYBR Green I detection to determine accurately the gene copy number of low copy genes in insect samples. The technique has overcome some problems inherent in other techniques: needs small amounts of sample (10 to 20 pg of DNA template are enough and that can be easily obtained from just one insect thorax), discriminates among low copy gene numbers (1 from 2, 3 or 4 copies) even in populations with high allelic variability (which it is very difficult with conventional Southern-blot technique or even with the qPCR TaqMan probes detection) and can detect tandem duplications of highly similar sequences (which it is not possible by Southern-blot or FISH techniques). Moreover, this technique is easier (i.e. not cytological information is necessary and there is no need of cytological preparations as in FISH), faster (i.e. Southern-blot or FISH are much time consuming techniques) and inexpensive (i.e. not target specific labeled probes are required as for qPCR with TaqMan chemistry detection).

## Conclusions

In the present work, qPCR with SYBR Green I detection has been successfully applied to determine gene dosage of the *O. nubilalis cadherin *gene and the *D. melanogaster BarH1 *and *BarH2 *genes. The technique has shown to be specific and accurate enough to distinguish between one and two gene copies per haploid genome of genes with high allelic variability. Furthermore, it is sensitive enough to give reliable results with a minimum amount of sample (DNA from individual thoraxes) and to detect gene duplications in tandem.

## Methods

### Insect strains and colonies

*D. melanogaster *wild type strain (*Oregon-R, Or-R*) and the mutant strain *Bar *(*B^1^*) were obtained from the Bloomington Drosophila Stock Centre (Indiana University, USA) and maintained in the laboratory in cornmeal/yeast medium, at 25°C, with a 16:8 h photoperiod (light:dark).

The *O. nubilalis *FR colony was obtained from the INRA, France, and had been maintained for many years in the laboratory. Larvae were reared on an artificial maize diet with agar, wheat germ, yeast, ascorbic and benzoic acids, and vitamins [31]. The *O. nubilalis *PH colony was established from larvae collected in commercial green pepper greenhouses from Pilar de la Horadada, in southeastern Spain and maintained in our laboratory for two years. The M colony was established in April 2009 from wild insects from corn fields kindly supplied from the Centro de Investigaciones Biológicas in Madrid, Spain. Adults were fed with cotton wool soaked in a solution of 10% honey in water (v:v). Insects were grown in a controlled chamber at 25°C, with a 16:8 h photoperiod (light:dark) and 70% humidity.

### DNA isolation

DNA was extracted from adult insects using the DNeasy Tissue Kit (Qiagen GmbH, Hilden, Germany) according to the manufacturer's instructions. In the case of *D. melanogaster*, each sample consisted of DNA from either four whole males or four female thoraxes. In the case of *O. nubilalis*, samples consisted of DNA from the thorax of frozen adults. Use of thoraxes instead of whole bodies avoids the contribution of DNA from eggs in females.

The DNA concentration was analyzed by measuring the absorbance at 260 nm. The purity of the sample was tested by obtaining the 260/280 nm ratio.

### Quantitative Real-Time PCR

In *D. melanogaster*, the low variability internal reference gene *RpS3 *[GenBank: NM057284] and the sex-linked genes *Bar-H1 *[GenBank: AY058309; FlyBase ref. CG5529, FBgn0011758] and *Bar-H2 *[GenBank: BT022144; FlyBase ref. CG5488, FBgn0004854] were selected for the analysis. In *O. nubilalis*, the selected genes were: *RpS3 *[GenBank: DQ988989] as internal reference gene, the *tpi *[GenBank: EU532457] and *ldh *[GenBank: EU532460] genes, both located in the Z chromosome [28], and the *cadherin *gene [GenBank: DQ000165] as an unknown copy number gene.

Primers were designed with the Primer Express Program (V 2.0.0, Applied Biosystems). The suitability of the primer pairs was verified *in silico *using the IDT Oligo Analizer (Integrated Device Technology, Inc, CA, USA) or the Genosys OligoMail ver. 2.0 (Genosys, Sigma-Aldrich, TX, USA) programs. For each target gene, several primer pairs were selected. Primers were provided by Sigma Life Science (Sigma-Aldrich).

The experimental suitability of each primer pair was verified by means of non-template control samples (to check primer-dimer formation in the experimental conditions), the *Ct *comparison with other primer pairs that amplify the same target gene, and with the dissociation curves of the amplicons generated in the experimental samples. The non-specific incorporation of the SYBR Green I dye into double-stranded DNA may cause an increase in the fluorescence reading due to amplification of non-specific products. Therefore, a final dissociation step was always performed at the end of each PCR assay to verify the unique and specific amplification of the target sequence. Table 1 summarizes the primers used in the present study.

**Table 1:** Primers used for the qPCR amplifications.

Insect	Target gene	Primer pair	Primer name	Sequence (5'→3')
*D. melanogaster*	*RpS3*	s3-1	s3-1 F	TCTTTCTTTTCTGCGCACCA
			s3-1 R	TCGCATTCATTTTGACGTCG
	
	*BarH1*	h1-4	h1-4 F	CCAGGACGATCCGTTGACA
			h1-4 R	GATCTGATCCTCGTCGTCCG
		h1-5	h1-5 F	TCCAGGTGTGAGCGGTACG
			h1-5 R	ATTAGCACACGCACACAATCG
	
	*BarH2*	h2-4	h2-4 F	CAGAACCAAATGGAAGCGTCA
			h2-4 R	TCGGCCAGCAGTTCCAAG
		h2-7	h2-7 F	GGAGGAACTGGCCCTGGA
			h2-7 R	GGAGGGTTGAATTCTCTCGGA

*O. nubilalis*	*RpS3*	s3-1	s3-1 F	GAGCTACTGGGAGAGAAGG
			s3-1 R	GATGTTGAAACGCTTCTGGA
		s3-2	s3-2 F	GCGTTTCAACATCCCTGAACA
			s3-2 R	GGCGACTTTCTCAGCGTACAG
	
	*tpi*	t1	t1 F	GGCGACAAGAATCAAATCAATG
			t1 R	AGGACCCTTTTTCAGAGTGTTCAC
	
	*ldh*	l1	l1 F	GAATAAATCGGGCTCGAAGGAC
			l1 R	TCACGCGAGACAGCTTCAAA
	
	*cadherin*	e26	e26F	ACGGCAACAACGAGGGTCT
			e26R	GAGATGACGTTGCGCGACT
		e28	e28F	CGAGCCACACAGAAGACGAC
			e28R	TGCTCGCACGGTCTATGATG

The letters F or R at the end of the primer names refer to their respective orientation (F = forward; R = reverse). The qPCR product sizes were 51 base pairs in all reactions except for primer pairs s3-1 and e26 of *O. nubilalis*, with lengths of 70 and 52 base pairs respectively.

The amplification reactions were run at least in triplicate. Reactions were performed in MicroAmp 96 well plates (Applied Biosystems) and contained 5 μl DNA, 3 μl of primers mix (final primer concentration of 300 nM each), and 12.5 μl of Power SYBR Green PCR Master Mix (Applied Biosystems), in a final volume of 25 μl. Amplifications were carried out in an ABI PRISM^® ^7000 Sequence Detection System or in a StepOne™ Real-Time PCR System (both from Applied Biosystems). To activate the Taq Polymerase, reactions started with two initial steps of 50°C for 2 min and 95°C for 10 min or with a single initial step of 95°C for 10 min depending on the detector used (ABI PRISM^® ^7000 Sequence Detection System or StepOne™ Real-Time PCR System, respectively). The reaction proceeded with 40 cycles of 95°C for 15 seconds and 60°C for 1 min for gene amplification. A final dissociation step was always performed to obtain the melting curves (thermal profile) of the amplicons obtained in the reactions.

Due to the genetic variability observed in some of the genes studied, to accurately estimate the gene dose we used at least two sets of primer pairs for each gene, and tested several samples. Moreover, insects from two to three different populations of *O. nubilalis *were tested.

### Data analysis

The qPCR technique requires that the amplification efficiencies of each one of the studied genes have to be close to 100% to allow comparison and joint analysis of the results. Amplification efficiencies were obtained using serial dilutions of the DNA samples with each one of the primers, and plotting the *C*t values obtained ("threshold cycle", i.e., the PCR cycle in which a significant increase of the amplified product is first detected) *versus *the DNA concentration. The efficiencies were calculated according to the equation *E *= [10^(-1/slope) ^- 1] × 100, where *E *= 100 corresponds to 100% efficiency. The *E *values obtained for all genes and primer pairs ranged from 97% to 103%, and, therefore, there was no need for any correction factor in the calculations of gene dosage [32,33].

Relative gene copy numbers (gene dose ratio, GDR) were obtained using the ΔCt method of relative quantification based on the fact that the genes under study (as well as their internal control gene) are amplified with the same efficiency in each sample. The relative gene copy number is calculated as 2^-ΔCt ^, where ΔCt = *Ct*_target _-*Ct*_reference _. The autosomal single copy gene *RpS3 *was used as a reference gene, so any autosomal single copy gene tested should give a GDR = 1 for both sexes, and an autosomal duplicated gene should give a GDR = 2 for both sexes. GRD values expected for genes located on sex chromosomes can be deduced in the same way, just taking into account the number of sex chromosomes. Lepidopterans have a female-heterogametic sex chromosome system (ZZ male and ZW female) in contrast to *D. melanogaster *in which the heterogametic sex is the male (XY male and XX female). Then, an X-located gene in *D. melanogaster *should have a GDR = 1 in females and 0.5 in males, whereas a Z-located gen in *O. nubilalis *should have a GDR = 1 in males and 0.5 in females.

## Competing interests

The authors declare that they have no competing interests.

## Authors' contributions

YB and BE conceived the study. YB, BE and JF participated in the experimental design and the writing of the manuscript. YB carried out the experimental work and the analysis of the data. All authors read and approved the final manuscript.
